# Mobilitätsangebote in der hausärztlichen Versorgung: Ein Überblick bestehender Angebotstypen und Potenziale für das deutsche Gesundheitswesen

**DOI:** 10.1055/a-2800-6420

**Published:** 2026-06-12

**Authors:** Oliver Legler, Nina Ratschker, Verena Maier, Jule Hofmann, Thomas Ewert, Gunnar Geuter

**Affiliations:** 1 Sachgebiet GP3 (Bayerische Gesundheitsagentur, Gesundheitsversorgung), Bayerisches Landesamt für Gesundheit und Lebensmittelsicherheit, Nürnberg, Germany

**Keywords:** Primärversorgung, mobile Versorgungseinrichtung, Kommune, ländliche Regionen, innovative Versorgungsmodelle, primary care, mobile health unit, municipality, rural regions, innovative health care models

## Abstract

**Hintergrund:**

Aktuelle Entwicklungen in Deutschland führen zu Konzentrationsprozessen hausärztlicher Versorgungsangebote, aus denen insbesondere in ländlich geprägten Räumen längere Wegstrecken oder -zeiten resultieren können. Zwar existieren bislang lediglich Empfehlungen zur Zumutbarkeit des Aufwands zu ihrer Erreichbarkeit. Gleichwohl werden vermehrt Anstrengungen, z. B. für mobilitätseingeschränkte Menschen und chronisch Erkrankte gefordert, um mögliche Zugangsbarrieren abzubauen. Ein Diskussionsstrang der letzten Jahre ist die Weiterentwicklung zugehender und aufsuchender hausärztlicher Versorgungsangebote. Dabei werden u. a. mobile Hausarztpraxen sowie spezifische Beförderungsangebote für Patientinnen und Patienten als Ergänzung zum ÖPNV und Individualverkehr diskutiert. Bedarfe und Stellenwert entsprechender Angebote im deutschen Gesundheitssystem wurden jedoch bislang kaum untersucht. Ziel dieser Arbeit ist es, einen Überblick über bestehende Angebotstypen zu geben, diese zu diskutieren und mögliche Potenziale für das deutsche Gesundheitswesen aufzuzeigen.

**Methodik:**

Neben Datenbankrecherchen zur Identifizierung wissenschaftlicher Publikationen wurde eine Internetrecherche durchgeführt. Es wurden sowohl nationale als auch internationale Ergebnisse berücksichtigt.

**Ergebnisse:**

Die identifizierten Ansätze aus den 21 einbezogenen Publikationen wurden nach Angebotstypen geclustert. Es wurden die vier Cluster „Eigenständige mobile Arztpraxis“, „Mobile Zweigpraxis“, „Spezifische Beförderungsangebote für Patientinnen und Patienten als Ergänzung des Nahverkehrs“ und „Maklerleistungen zur Beförderung von Patientinnen und Patienten“ herausgearbeitet. Bei Konzeption und Implementierung förderlich, hat sich v. a. ein eng aufeinander abgestimmtes Vorgehen relevanter Akteure mit partizipativem Ansatz zur Berücksichtigung verschiedener Perspektiven gezeigt. Hemmend wirken neben rechtlichen Hürden v. a. nicht kostendeckende Wirtschaftlichkeitsaspekte, die oft mit der eher geringen Inanspruchnahme der Angebote assoziiert sind.

**Schlussfolgerung:**

Mobile Arztpraxen und Angebote zur Ergänzung bestehender Mobilitätsoptionen für Patientinnen und Patienten können potenziell einen Beitrag zum Abbau von Zugangsbarrieren zu einer wohnortnahen hausärztlichen Versorgung leisten, insbesondere wenn sie Teil eines Gesamtkonzeptes für eine multiprofessionelle Primärversorgung sind und die jeweiligen Bedarfslagen berücksichtigen. Zur Konkretisierung des Potenzials ist eine wissenschaftlich fundierte Evaluation etwaiger, weiterer Modellprojekte empfehlenswert. Dabei sollten regionale Bedarfe und Bedürfnisse konsequent berücksichtigt werden.

## Abstract (en)

**Background:**

Current developments in Germany are leading to concentration processes of primary care services, which can result in longer distances or travel times, especially in rural areas. So far, there are only recommendations regarding the reasonableness of the effort to reach them. Nevertheless, there are increasing calls for efforts, for example, for people with limited mobility and chronic illnesses, to reduce potential access barriers. One discussion thread in recent years has been the further development of proactive and outreach primary care services. This includes discussions about mobile GP practices as well as specific transportation offers for patients to complement public transport and individual travel. However, the needs and significance of such offerings in the German healthcare system have hardly been investigated so far. The aim of this paper was to provide an overview of existing types of offerings, discuss them and highlight their potential for the German healthcare system.

**Methods:**

In addition to searching data banks to identify scientific publications, an internet search was conducted. Both national and international results were taken into consideration.

**Results:**

The identified approaches from the 21 included publications were clustered according to their types of offerings. The four clusters “Independent mobile doctor’s practice”, “Mobile branch practice”, “Specific transportation offers for patients as a complement to local transport” and “Brokerage services for patient transportation” were identified. A closely coordinated approach of relevant actors with a participatory approach to consider different perspectives was proved to be beneficial in conception and implementation. In addition to legal hurdles, non-cost-covering economic aspects, often associated with the rather low use of the offers, proved to be inhibiting.

**Conclusion:**

Mobile doctor’s offices and offers to supplement existing mobility options for patients can potentially reduce access barriers to local primary care, especially if they are part of an overall concept for multidisciplinary primary care and take into account the respective needs. To concretize the potential, scientific evaluation of pilot projects is recommended. Regional needs and requirements should be consistently taken into account.

## Hintergrund und Zielstellung der Arbeit

Die medizinische Versorgung in Deutschland steht vor großen Herausforderungen: So führen mit dem demographischen Wandel assoziierte Auswirkungen auf die Angebots- und Nachfragestruktur medizinischer Leistungen 1 2 3 , medizinisch-technischer Fortschritt und gesamtgesellschaftliche Entwicklungen u. a. zu Konzentrationsprozessen 4 5 6 , welche voraussichtlich noch zunehmen werden, beispielsweise im Zuge der Entwicklungen von (Primär-)Versorgungszentren (ex. 2 3 6 7 8 9 ). Besonders für ländliche Räume bedarf es vor diesem Hintergrund neuer Strategien und angepasster Rahmenbedingungen 2 3 6 . Aus gesundheitsplanerischer Perspektive ist auch darauf abzustellen, dass Einrichtungen für Bürgerinnen und Bürger in zumutbarem Maße erreichbar bzw. zugänglich sind 10 .

In Deutschland werden Diskussionen zur Erreichbarkeit und Zugänglichkeit überwiegend entlang der hausärztlichen Versorgung geführt 2 . In Ergänzung bereits bewährter 11 „neuerer“ (z. B. Bürgerbus oder Ruftaxi) und „klassischer“ (z. B. Linienbus) Formen des ÖPNV sowie des Individualverkehrs werden zum einen spezifische Beförderungsangebote für Patientinnen und Patienten wie gesonderte Hol- und Bringdienste 6 und zum anderen Angebote der aufsuchenden Gesundheitsversorgung wie mobile hausärztliche Praxen 2 3 12 diskutiert.

Allerdings mangelt es bisher sowohl zu spezifischen Beförderungsgeboten zur besseren Erreichbarkeit hausärztlicher Versorgung als auch zu aufsuchenden Angeboten für Deutschland an Erfahrungen 12 . Auch fehlt ein Überblick bestehender Angebote und deren Potenziale. Ein entsprechender Überblick inklusive Diskussion und Ableitung möglicher Potenziale für das deutsche Gesundheitswesen ist Ziel dieser Arbeit.

## Methodik

Mit Stichtag zum 26.08.2020 wurde eine Literaturrecherche in den Datenbanken PubMed und Cochrane Library durchgeführt. Eine ergänzende Suche wurde für den Zeitraum vom 27.08.2020 bis einschließlich 20.06.2023 in PubMed umgesetzt. Für die Literaturrecherche in den Datenbanken wurden folgende MeSH Terms verwendet: „Mobile Health Units“; „Transportation of Patients“; „Health Services Accessibility“; „Delivery of Health Care“; „Rural Health Services“; „Rural Health“. Es wurden ausschließlich Werke in deutscher und englischer Sprache mit Verfügbarkeit eines Abstracts berücksichtigt. Die Gesamtzahl der anhand der aufgeführten Kriterien identifizierten Abstracts belief sich auf 2.623. Zudem wurden mittels Internetrecherche weitere wissenschaftliche Publikationen sowie graue Literatur (z. B. Kongressbeiträge, Präsentationen etc.) aus dem deutschsprachigen Raum einbezogen. Auf Basis der Internetrecherche konnten 12 weitere relevante Publikationen identifiziert werden. Nach Ausschluss von Duplikaten belief sich die Gesamtzahl an Publikationen auf 2.577.

Im Rahmen des Reviews wurden Angebote des stationären Sektors und des Rettungswesens sowie rein pflegerische Angebote ausgeschlossen. Zudem wurden Modelle mit Präventionsschwerpunkt bzw. Angebote zum Screening, zahnärztliche Angebote und reine Telemedizin- bzw. Delegationsmodelle ebenso nicht berücksichtigt wie jene Angebote, die spezifische Zielgruppen und/oder Erkrankungsbilder fokussieren. Ausgeschlossen wurden darüber hinaus Ansätze mit sehr geringem Übertragungspotenzial auf Deutschland bzw. das deutsche Gesundheitswesen (z. B. „Flying Doctors“ aus Australien oder Modelle aus Subsahara-Afrika zur Basisversorgung der Bevölkerung).

Eingeschlossen wurden demgegenüber Publikationen aus dem Bereich „primary care“, die dem Leistungsspektrum der hausärztlichen Versorgung gemäß „Bundesmantelvertrag – Ärzte“ (BMV-Ä) zugeordnet werden können. Dieses umfasst u. a. die Durchführung und Veranlassung diagnostischer, therapeutischer sowie präventiver Maßnahmen und umschreibt die koordinierende Funktion der Hausärztinnen und Hausärzte 13 . Hinsichtlich der spezifischen Beförderungsangebote für Patientinnen und Patienten wurden ausschließlich Angebote aufgenommen, welche explizit dem Zweck der Beförderung zu ärztlichen Einrichtungen dienen.

**Abb. 1:**
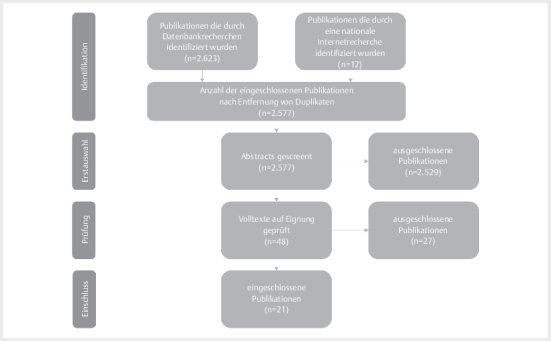
Flow-Diagramm zum Prozess der Auswahl an eingeschlossenen Publikationen.

Während des Reviewprozesses haben drei Reviewerinnen bzw. Reviewer zunächst 100 Artikel gemeinsam gesichtet, die Auswahlkriterien überprüft und geschärft. Im Anschluss haben zwei Reviewerinnen bzw. Reviewer unabhängig voneinander 20% der identifizierten Publikationen aus der Datenbankrecherche gesichtet und eine Auswahl an Publikationen auf Abstractebene durchgeführt. Die Übereinstimmungsquote lag hierbei bei 98%. Im Zweifelsfall wurde eine dritte Person hinzugezogen. Die Auswahl aller weiteren Publikationen wurde von einem Reviewer durchgeführt. Nach Sichtung aller Publikationen auf Abstractebene wurden 48 Publikationen in die Volltextanalyse einbezogen. Die Auswahl auf Volltextebene ergab 21 relevante Publikationen für die eingangs beschriebene Fragestellung (vgl. Abb. 1 ).

## Ergebnisse

Insgesamt wurden 21 Publikationen einbezogen. Anhand ihrer Angebotstypen wurden die identifizierten Ansätze mittels induktiven Verfahrens geclustert. Es wurden die vier Cluster „Eigenständige mobile Arztpraxis“, „Mobile Zweigpraxis“, „Spezifische Beförderungsangebote für Patientinnen und Patienten als Ergänzung des Nahverkehrs“ und „Maklerleistungen zur Beförderung von Patientinnen und Patienten“ herausgearbeitet.

### Eigenständige mobile Arztpraxis

Ansätze, die diesem Cluster zugeordnet wurden, erfüllten die nachfolgenden Kriterien:

- Neugründung einer mobilen Hausarztpraxis
- eigenständige Praxis, unabhängig von bestehenden Praxen in der Region
- umfassendes Leistungsspektrum vergleichbar gemäß BMV-Ä 13

Es wurden zwei, im Grundsatz vergleichbare Ansätze identifiziert. Bei beiden, in Deutschland erprobten mobilen Hausarztpraxen, handelt es sich jeweils um eine Eigeneinrichtung der Kassenärztlichen Vereinigung (KV) Hessen bzw. KV Niedersachsen 14 15 16 in umgebauten Nutzfahrzeugen.

Der technische Umbau wurde durch Kooperationen mit Projektpartnern aus der Privatwirtschaft realisiert 14 15 . Ziel war es, eine Entlastung der Hausarztpraxen in den jeweiligen Regionen zu erreichen, für die im Vorfeld Versorgungsdefizite identifiziert wurden. Die sorgfältige und kontinuierliche Analyse der Ausganglage unter Einbindung der regionalen Ärzteschaft, der Kommunen und anderer Interessensgruppen diente auch dazu, Konkurrenz zu etablierten Einrichtungen zu vermeiden 15 . Die kommunale Beteiligung bei der Einrichtung (z. B. über die Bereitstellung von Warteräumen und sanitären Anlagen) wurde als eine relevante Unterstützungsleistung beschrieben 15 . Als diffizil haben sich rechtliche Fragestellungen (z. B. aus Berufs- und Vertragsarztrecht) erwiesen 15 . Ein wirtschaftlicher Betrieb zeigte sich in der Umsetzung beider Ansätze als schwierig. Den im Vergleich zu herkömmlichen Praxen höheren Aufwendungen stand eine geringere Auslastung gegenüber. Im niedersächsischen Projekt konnten z. B. maximal 59% Auslastung einer durchschnittlichen Praxis erreicht werden, was als ein Grund für die Einstellung nach Projektende angeführt wurde 15 . Die mobile Hausarztpraxis in Hessen wird mit finanzieller Unterstützung durch öffentliche Gelder weiter betrieben 16 17 .

### Mobile Zweigpraxis

Diesem Cluster wurden Ansätze zugeordnet, die die nachfolgenden Kriterien erfüllten:

- Bildung einer mobilen, hausärztlichen Zweig-/ bzw. Filialpraxis
- organisatorische Anbindung an bereits in der Region bestehende Praxen

Es konnte ein in den 1980er Jahren erprobter Ansatz aus Großbritannien identifiziert werden 18 , der, auf das deutsche Gesundheitswesen übertragen, vertragsarztrechtlich als Zweigpraxis eingeordnet werden könnte. Zweig-/ bzw. Filialpraxen sind im deutschen Recht keine eigenständigen, neuen Praxen, sondern Genehmigungen eines zusätzlichen Tätigkeitsorts einer Vertragsärztin bzw. eines Vertragsarztes 19 .

Beim identifizierten Ansatz handelte es sich um ein Gemeinschaftsprojekt von vier Hausärzten aus der dünn besiedelten Grafschaft Norfolk, die bis dato zwei Hauptstandorte und mehrere, stundenweise geöffnete Zweigpraxen betrieben 18 . Als eine der Zweigpraxen mangels zur Verfügung stehender Räumlichkeiten geschlossen werden musste, nutzten die Ärzte zur Aufrechterhaltung des Versorgungsangebotes in der Fläche stattdessen einen mit einfachen medizinischen Geräten ausgestatteten Wohnwagen, der von einem PKW gezogen wurde 18 . Das im Vergleich zu einer „klassischen“ Hausarztpraxis eingeschränkte Angebotsspektrum umfasste grundlegende ärztliche Beratungen und Untersuchungen 18 . Das Projekt wurde durch Spenden lokaler Unternehmen und Banken finanziell ermöglicht. Das nationale Gesundheitsministerium lehnte eine Kostenübernahme, u. a. aus abrechnungstechnischen und erwarteten, unvorteilhaften Kosten-Nutzen-Aspekten, ab 18 .

Ein großer Teil der Patientinnen und Patienten der vormals herkömmlichen Filiale nutzte auch das mobile Angebot. Im Bereich der Beratungsleistungen konnte ein Anstieg verzeichnet werden 18 . Da die basale technische Ausstattung nur eingeschränkt Untersuchungen und Behandlungen ermöglichte, konnte jedoch keine tatsächliche Entlastung der Hauptpraxen festgestellt werden 18 . Hinsichtlich der Wirtschaftlichkeit solcher Praxen vermuteten die Autoren potenzielle Vorteile gegenüber herkömmlichen Filialen aufgrund geringerer Fixkosten, wenngleich im Projekt keine dezidierte wirtschaftliche Analyse durchgeführt wurde 18 .

### Spezifische Beförderungsangebote für Patientinnen und Patienten als Ergänzung des Nahverkehrs

Diesem Cluster wurden Ansätze zugeordnet, die die nachfolgenden Kriterien erfüllten:

- kein Teil des ÖPNV
- spezifische Beförderungsangebote zu ambulanten Einrichtungen vergleichbar dem Leistungsspektrum der hausärztlichen Versorgung gemäß BMV-Ä 13
- Beförderung von Personen, die sich nicht in einer Notfallsituation befinden

Im angloamerikanischen Raum sind derartige Dienstleistungen als „Non-Emergency Medical Transportation“ (NEMT) bekannt und in zahlreichen ländlichen Regionen mittlerweile etabliert (ex. 20 21 22 ). Teilweise werden eigene Busrouten eingerichtet, die u. a. Einrichtungen vergleichbar dem Leistungsspektrum der hausärztlichen Versorgung gemäß BMV-Ä 13 anfahren. Einige Programme stehen besonders hilfsbedürftigen Personengruppen als soziale Unterstützungsleistung offen, andere können von allen Bürgerinnen und Bürgern genutzt werden 21 . Die Beförderungsangebote konnten u. a. durch Subventionierung (öffentliche Mittel) sowie das Anfahren mehrerer, auch größerer, hochfrequentierter Einrichtungen in der Regel langfristig etabliert werden und werden von der Bevölkerung gut angenommen (ex. 20 21 22 ). Die Studienlage zeigt, dass der Einsatz eigens gestellter, regelmäßig verkehrender Fahrzeuge zielführend sein kann, wenn erstens sehr große Einzugsgebiete abgedeckt werden müssen und zweitens große Einrichtungen wie Krankenhäuser oder Gesundheitszentren angesteuert werden 20 21 .

Im deutschen Raum konnte ein vergleichbarer Ansatz identifiziert werden: In Brandenburg (Landkreis Märkisch-Oderland) wurde zwischen 2012 und 2013 von der KV Brandenburg, in Kooperation mit Arztpraxen, Krankenkassen, Kommunen und Verkehrsbetrieben, ein unabhängig vom ÖPNV verkehrender Patientenbus getestet. Ziel des kommunal finanzierten Projekts war eine verbesserte Erreichbarkeit der Gesundheitsversorgungsangebote, u. a. der hausärztlichen Versorgung, für Bürgerinnen und Bürger sowie eine Entlastung der niedergelassenen Ärzteschaft von Hausbesuchen 23 . Das spezifische Beförderungsangebot wurde nach der Projektphase eingestellt. Einerseits aufgrund einer insgesamt zu geringen, nicht wirtschaftlich tragfähigen Auslastung. Andererseits nahm ein Großteil der Nutzerinnen und Nutzer das Angebot letztlich für andere Zwecke wahr, etwa um Einkäufe zu tätigen 23 24 . Dennoch wurde der kooperative Planungs- und Umsetzungsprozess als beispielgebend für die Entwicklung künftiger Lösungsansätze beschrieben 23 24 . Aufgrund der gesammelten Erfahrungen wurde der Patientenbus im Anschluss als kommunal geführtes Bürgerbus-Modell „zweckoffen“ fortgeführt 23 .

### Maklerleistungen zur Beförderung von Patientinnen und Patienten

Diesem Cluster wurden Ansätze zugeordnet, die die nachfolgenden Kriterien erfüllten:

- kein Teil des ÖPNV
- Nutzung bereits bestehender Beförderungsangebote
- Übernahme von Maklerleistungen für Patientinnen und Patienten zur Beförderung zu ambulanten Einrichtungen vergleichbar dem Leistungsspektrum der hausärztlichen Versorgung gemäß BMV-Ä 13

Maklerleistungen zur Beförderung von Patientinnen und Patienten werden v. a. in den USA vielfach genutzt 25 26 . Die jeweilige Ausgestaltung unterscheidet sich teilweise stark. In einigen Fällen schließen Betreiber gesonderte Verträge mit Beförderungsunternehmen ab 25 26 .

Hierfür konnte auch ein Ansatz aus Deutschland identifiziert werden: In Bayern (Kreisregion Coburg) wurde ein Hol- und Bringdienst im sektorenübergreifenden Netzwerk der Gesundheitsregion ^plus^ entwickelt, um Patientinnen und Patienten ohne eigene Fahrgelegenheit zu einem geringen Eigenbeitrag per Taxi zur regionalen Bereitschaftspraxis zu befördern. Die Kommunen kooperieren hierbei mit einem lokalen Taxiunternehmen und erhalten finanzielle Unterstützung eines ansässigen Wirtschaftsunternehmens 27 . Das Angebot besteht seit 2015 28 .

In einigen internationalen Ansätzen wurden Kooperationen mit Taxiunternehmen für Fahrten zu einzelnen Praxen mangels Inanspruchnahme wieder eingestellt 26 . Forschende sahen die letztlich sehr kleine Zielgruppe von Personen ohne Möglichkeiten einer Nutzung von PKW oder ÖPNV ursächlich und empfahlen bei der Ausgestaltung solcher Unterstützungsangebote verstärkt auf Bedarfsgerechtigkeit zu achten 26 .

Des Weiteren werden international Lösungen erprobt, in denen Gesundheitseinrichtungen bzw. Hausarztpraxen ihren Patientinnen und Patienten lediglich die Übernahme der Buchung bei einem unabhängigen Beförderungsdienstleistenden anbieten. In den USA offerieren vermittelnde Online-Plattformen wie Uber und Lyft Buchungsmöglichkeiten und haben sich bereits fest im NEMT-Markt etabliert. Die Plattformen bieten teilweise für Praxen entwickelte Bedienoberflächen zur Berücksichtigung spezifischer Anforderungen (z. B. Fahrzeug mit Rampe für Menschen im Rollstuhl) 22 25 29 . Insbesondere Patientinnen und Patienten, die sich mangels technischer Möglichkeiten oder Kompetenzen (z. B. kein Smartphone, Sprachbarrieren) nicht selbst kümmern können, soll hierdurch der Zugang zu ärztlichen Leistungen erleichtert werden. Praxen erhoffen sich eine bessere Planbarkeit und zuverlässigere Terminwahrnehmung 22 30 . Die Angebote werden oft langfristig angenommen, wenn Buchungsmöglichkeiten vom Praxispersonal effizient genutzt werden können und die Kosten von Patientinnen und Patienten als akzeptabel erachtet werden. Grundvoraussetzung ist zudem, dass in der jeweiligen Region überhaupt Online-Plattform-basierte Angebote verfügbar sind 22 .

## Diskussion

Es existieren vielfältige Ansätze zur Förderung der Erreichbarkeit und zum Abbau von Zugangsbarrieren zu (haus-)ärztlichen Leistungen. Der Überblick zeigt, dass international erprobte Ansätze technisch und organisatorisch gesehen prinzipiell im deutschen Gesundheitssystem ebenfalls umsetzbar wären. Eine Unterstützung seitens der öffentlichen Hand und ggf. auch der Privatwirtschaft hat sich als förderlich erwiesen. National gibt es diesbezüglich jedoch noch vergleichsweise wenig Erfahrungen.

Grundsätzlich zeigt sich sowohl international als auch national, dass die Planung und etwaige Ausgestaltung stark von der regionalen Ausgangslage abhängig gemacht werden sollte. Frühzeitig und unter Einbindung der relevanten lokalen Akteure sollten Bedarfslagen und Bedürfnisse identifiziert und analysiert werden. Wie häufig bei neuartigen Ansätzen zu beobachten, erweisen sich zudem rechtliche Rahmenbedingungen regelmäßig als potenzielle Hürde. Im nationalen Kontext ist es deshalb zu empfehlen, mögliche rechtliche Implikationen frühzeitig im Planungsprozess gemeinsam mit den jeweils Zuständigen wie Ärztekammern, Kassenärztlichen Vereinigungen oder, etwa bei Subventionstatbeständen aus öffentlicher Hand, der Kommunalaufsicht zu klären.

Bei der Implementierung eigenständiger mobiler Hausarztpraxen scheint aufgrund vergleichsweise hoher Kosten eine unabhängige, wirtschaftliche Tragfähigkeit nur schwer erzielbar. Den Kosten könnten allerdings gesamtgesellschaftliche Ersparnisse, z. B. aufgrund vermiedener Krankenhauseinweisungen gegenübergestellt werden 31 . Konstatiert werden muss eine deutlich geringere Auslastung mobiler Praxen im Vergleich zu herkömmlichen Praxen, obwohl diese in Regionen eingesetzt wurden, in denen Indikatoren und Vorstudien auf einen erhöhten Bedarf an zusätzlichen Leistungsangeboten für die hausärztliche Versorgung hindeuteten. Dies wirft wiederum die Frage auf, inwiefern bestehende Mobilitätsangebote bedarfsgerechte und nutzerorientierte Lösungen darstellen. Denn Bedarfe und Bedürfnisse sind vielschichtig: Nicht nur bei älteren, chronisch Kranken spielen neben medizinischen Kompetenzen der Behandelnden weitere Faktoren wie die faktische Verfügbarkeit (z. B. tatsächliche Erreichbarkeit des Angebots für mobilitätseingeschränkte Personen oder bei Akuterkrankungen „kompatible“ Öffnungszeiten) eine Rolle. Auch psychosoziale Komponenten sind nicht zu unterschätzen. Diese äußern sich z. B. im Wunsch nach kontinuierlicher Behandlung durch vertrautes Personal oder im Wunsch nach Versorgung im häuslichen Umfeld 32 33 .

Die Behandlung durch vertrautes Personal könnte ein Potenzial mobiler Zweigpraxen von, in der Region bereits ansässigen Hausarztpraxen darstellen. Das identifizierte Beispiel aus Großbritannien gibt dazu erste Hinweise 18 , wenngleich hierbei zu berücksichtigen ist, dass das Beispiel bereits vor mehreren Jahrzehnten erprobt wurde und keine aktuelleren Fallbeispiele bzw. Erfahrungswerte identifiziert werden konnten. Unabhängig davon könnten aus wirtschaftlicher Sicht, je nach Konstellation, mobile Zweigpraxen gegenüber herkömmlichen Zweigpraxen in Immobilien Vorteile bieten. In Deutschland plante beispielsweise eine Privatpraxis aus Niedersachsen mit Unterstützung einer Kommune und Fördergeldern der EU einen Umbau eines ehemaligen Zirkuswagens, um hausärztliche Leistungen im ländlichen Raum anzubieten. Auch aufgrund berufsrechtlicher Fragestellungen wurde eine Realisierung allerdings vorerst ad acta gelegt 34 35 .

Sowohl für eigenständige mobile Hausarztpraxen als auch mobile Zweigpraxen muss unabhängig davon die Frage nach einem effizienten Einsatz der knappen Ressource „Arztzeit“ 36 diskutiert werden. Denn: Ob mobile Zweigpraxen lokale Hausarztpraxen entlasten bzw. Ärztinnen und Ärzten in eigenständigen mobilen Praxen ihre Leistungen effizient anbieten können ist nicht abschließend klar. Gegebenenfalls könnte der Einsatz multiprofessioneller Teams, wie z. B. vom Sachverständigenrat für Gesundheit und Pflege empfohlen, einen Beitrag zur Lösung leisten 3 37 38 . Entsprechende Ansätze wären auch aus grundsätzlichen Überlegungen zur Weiterentwicklung der Versorgungsstrukturen in einer Gesellschaft des längeren Lebens und einer Zunahme altersassoziierter und chronischer Erkrankungen empfehlenswert 3 .

Spezifische Beförderungsgebote für Patientinnen und Patienten als Ergänzung des Nahverkehrs haben sich bislang in Deutschland nicht durchgesetzt. Demgegenüber erscheinen universell nutzbare Ruf- oder Bürgerbusse praktikabler. Ein Grund dafür ist, dass potenzielle Zielgruppen spezifische Beförderungsangebote nur selten nachfragen. Vielmehr benötigen sie in aller Regel einen entsprechenden Fahrdienst z. B. auch für den Besuch anderer Einrichtungen der Nahversorgung 23 . Statt spezifische Beförderungsangebote zu planen, legen die vorliegenden Ergebnisse den Schluss nahe, über „zweckoffene“, „verschränkte“ Beförderungsangebote in Ergänzung des Nahverkehrs nachzudenken. Eine auf die Verbesserung der Mobilität ausgerichtete Gesamtstrategie hätte im Sinne des Co-Benefits-Ansatzes Auswirkungen auf mehrere Zielbereiche, auch außerhalb des Gesundheitswesens. Nicht zuletzt könnten dadurch Effizienzpotenziale gehoben werden. Bei der Planung und Umsetzung derartiger Angebote sollte zudem bedacht werden, dass für mobilitätseingeschränkte Personen etwaige Fußwege zu festen Haltestellen ein unüberbrückbares Hindernis darstellen können 39 .

Auch individualisierte Maklerleistungen zur Beförderung von Patientinnen und Patienten könnten einen Beitrag zur besseren Erreichbarkeit hausärztlicher Versorgungsangebote leisten. Die Ergebnisse weisen im Sinne der Nutzerorientierung allerdings darauf hin, insbesondere bei der technischen Ausgestaltung auf Bedarfs- und Zielgruppengerechtigkeit zu achten 26 . Subventionen, vor allem bei der Entwicklung und Etablierung von Lösungen, erscheinen zudem ebenso notwendig, wie die Klärung möglicher Rechtsfragen, z. B. bei Nutzung plattformbasierter Dienstleistungsunternehmen wie Uber 40 . Die Nutzung bestehender Angebote lässt einen geringeren Implementierungsaufwand erwarten. Auch „Tür-zu-Tür“-Leistungen könnten relativ leicht umgesetzt werden. Bedacht werden sollten allerdings u. a. Abhängigkeiten von kommerziell ausgerichteten, nicht zur Dienstleistung verpflichteten Drittanbietern. Für diese sind ländliche Räume, die häufig ohnehin von sich wechselseitig verstärkenden infrastrukturellen und soziodemografische Negativentwicklungen betroffen sind 41 , aufgrund geringerer Nachfrage und langen Wegestrecken oft weniger lukrativ 42 . Diesem Umstand sollte u. a. aus Gründen der Zugangsgerechtigkeit im Sinne bedarfsgerechter Steuerung der Versorgung Rechnung getragen werden.

Insgesamt ist anzumerken, dass bezüglich der im Rahmen der vorliegenden Arbeit identifizierten Publikationen zu in Deutschland erprobten Ansätzen die Quellenlage kritisch zu hinterfragen ist: Nur in Ausnahmefällen wurden nach wissenschaftlichen Standards belastbare Evaluationen durchgeführt bzw. veröffentlicht. Ein großer Teil jener Quellen ist zudem der grauen Literatur zuzuordnen. Teilweise waren nach anfänglichen Veröffentlichungen zu (geplanten) Vorhaben keine Quellen zum weiteren Verlauf auffindbar. Dies deutet zum einen auf ein Publikationsbias oder zumindest Publikationsverzögerungen hin und wirkt sich zum anderen auf die Aussagekraft der aus der bestehenden Literatur ableitbaren Rückschlüsse aus. Dieses Problem scheint generell bei Projekten im Bereich der Mobilität in ländlichen Räumen zu bestehen. So fordert auch das Bundesinstitut für Bau-, Stadt- und Raumforschung von Bund und Ländern, den Fokus auf die Evaluation der Wirksamkeit und Effizienz von durchgeführten Modellprojekten zu legen anstatt auf die Generierung immer neuer Projekte 39 . Vor dem Hintergrund bisheriger Erfahrungen sollten dabei zum einen Bedarfs- und Bedürfnislagen berücksichtigt werden. Denn Nutzerorientierung ist ein entscheidender Faktor hinsichtlich Zufriedenheit und Akzeptanz von Patientinnen und Patienten und beeinflusst schlussendlich die Nachfrage nach Versorgungsangeboten 43 . Zum anderen sollten aufgrund ihres oben angesprochenen Einflusses regelhaft fördernde und hemmende Faktoren sowohl aus rechtlicher als auch wirtschaftlicher Sicht identifiziert werden.

## Limitationen

Trotz umfangreicher Recherchen sind die Ergebnisse und Interpretationen in dieser Arbeit durch einige Limitationen gekennzeichnet. So wurde zwar in wissenschaftlichen Datenbanken nach englisch- und deutschsprachigen Publikationen gesucht. Die Ergänzung anhand von grauer Literatur erfolgte jedoch nur für deutschsprachige Literatur. Folglich dürften Modelle aus dem deutschsprachigen Raum mit weniger valider Quellengrundlage überrepräsentiert sein. Die eher geringe Anzahl an wissenschaftlichen Veröffentlichungen deutet, wie bereits ausgeführt, zudem auf ein Publikationsbias hin. Eine Erklärung für ein solches Bias wäre, dass bei negativ verlaufenden Projekten oft auf weiterführende Publikationen verzichtet wird. Nichtsdestotrotz konnte festgestellt werden, dass die Schilderungen innerhalb der grauen Literatur nicht grundlegend von den Resultaten der wissenschaftlich ausgewerteten Modelle abweichen. Ein weiteres Bias kann sich durch das Auswahlkriterium der Publikationen aus dem Bereich „primary care“, die dem Leistungsspektrum der hausärztlichen Versorgung gemäß BMV-Ä 13 entsprechen, ergeben. Dieses Kriterium war nur in wenigen Quellen konkret auffindbar und wird in anderen Gesundheitssystemen unterschiedlich interpretiert. Die Auswahl erfolgte demnach teilweise anhand von Einschätzungen des jeweiligen Ansatzes. Nicht ausgeschlossen werden kann außerdem, dass durch die Fokussierung auf diesen Bereich relevante, ggf. auf die hausärztliche Versorgung übertragbare, Erkenntnisse aus anderen Publikationen nicht gefunden werden konnten. Des Weiteren ist einschränkend anzumerken, dass die Recherche bereits im Jahr 2023 abgeschlossen wurde und etwaige, neuere Modelle demnach nicht berücksichtigt werden konnten. Insgesamt ist dennoch davon auszugehen, dass die genannten Limitationen die Kernaussage der Arbeit nicht maßgeblich beeinflussen.

## Fazit

Zusammenfassend kann konstatiert werden, dass Mobilitätsangebote potenziell einen Beitrag zum Abbau von Zugangsbarrieren zur wohnortnahen hausärztlichen Versorgung leisten können und grundsätzlich auf Offenheit in der Bevölkerung stoßen 44 . Weitere erfolgreich verlaufende Modellprojekte könnten die bislang in Realitas oft geringe Nachfrage bzw. Akzeptanz 44 sowie die Wirtschaftlichkeit steigern. Zudem könnten sie wichtige Hinweise auf notwendige Handlungsbedarfe zur Weiterentwicklung rechtlicher Rahmenbedingungen liefern. Insofern werden die Ergebnisse aktuell laufender Projekte, wie das im Sommer 2024 von der KV Rheinland-Pfalz gestartete Vorhaben, bei dem speziell ausgestattete Fahrzeuge übergangsweise in Regionen mit kurzfristig entstandenen Versorgungsengpässen eingesetzt werden sollen 45 46 , mit Interesse zu verfolgen sein.

Bei der Konzeption von Mobilitätsangeboten sollte berücksichtigt werden, diese im Rahmen strategischer Gesundheitsplanung als Teil eines Gesamtkonzepts für eine multiprofessionelle Primärversorgung unter Berücksichtigung jeweiliger Bedarfslagen nutzerorientiert zu entwickeln. Zudem sollten die Angebote bestenfalls in eine übergreifende Mobilitätsstrategie eingebettet werden. Zuletzt sollten die maßgeblichen Akteure zur Berücksichtigung möglichst aller relevanten Perspektiven bei der Entwicklung beteiligt werden. Dies setzt regelhaft ein sektoren- und ressortübergreifendes Vorgehen voraus.
